# Recognition of HIV-1 Peptides by Host CTL Is Related to HIV-1 Similarity to Human Proteins

**DOI:** 10.1371/journal.pone.0000823

**Published:** 2007-09-05

**Authors:** Morgane Rolland, David C. Nickle, Wenjie Deng, Nicole Frahm, Christian Brander, Gerald H. Learn, David Heckerman, Nebosja Jojic, Vladimir Jojic, Bruce D. Walker, James I. Mullins

**Affiliations:** 1 Department of Microbiology, University of Washington, Seattle, Washington, United States of America; 2 Partners AIDS Research Center, Massachusetts General Hospital, Harvard Medical School, Charlestown, Massachusetts, United States of America; 3 Machine Learning and Applied Statistics Group, Microsoft Research, Redmond, Washington, United States of America; University of California at San Francisco, United States of America

## Abstract

**Background:**

While human immunodeficiency virus type 1 (HIV-1)-specific cytotoxic T lymphocytes preferentially target specific regions of the viral proteome, HIV-1 features that contribute to immune recognition are not well understood. One hypothesis is that similarities between HIV and human proteins influence the host immune response, i.e., resemblance between viral and host peptides could preclude reactivity against certain HIV epitopes.

**Methodology/Principal Findings:**

We analyzed the extent of similarity between HIV-1 and the human proteome. Proteins from the HIV-1 B consensus sequence from 2001 were dissected into overlapping k-mers, which were then probed against a non-redundant database of the human proteome in order to identify segments of high similarity. We tested the relationship between HIV-1 similarity to host encoded peptides and immune recognition in HIV-infected individuals, and found that HIV immunogenicity could be partially modulated by the sequence similarity to the host proteome. ELISpot responses to peptides spanning the entire viral proteome evaluated in 314 individuals showed a trend indicating an inverse relationship between the similarity to the host proteome and the frequency of recognition. In addition, analysis of responses by a group of 30 HIV-infected individuals against 944 overlapping peptides representing a broad range of individual HIV-1B Nef variants, affirmed that the degree of similarity to the host was significantly lower for peptides with reactive epitopes than for those that were not recognized.

**Conclusions/Significance:**

Our results suggest that antigenic motifs that are scarcely represented in human proteins might represent more immunogenic CTL targets not selected against in the host. This observation could provide guidance in the design of more effective HIV immunogens, as sequences devoid of host-like features might afford superior immune reactivity.

## Introduction

HIV-1-specific CD8+ T cell responses play a pivotal role in controlling HIV-1 replication, despite being rarely if ever able to fully contain viral replication *in vivo*. The protective role of CD8+ T cells against disease progression is strongly suggested by studies in HIV-infected individuals [1]–[3] and by T cell depletion studies in SIV-infected rhesus macaques [4]–[6]. However, definition of the specificity and functional characteristics of the CD8+ T cell-mediated immunity required for effective viral control has proven elusive.

Considering the high number of potential epitopes, anti-HIV CTL responses tend to target a relatively limited set of epitopes in a distinct hierarchical pattern [7], with some epitopes engendering strong responses (immunodominant), weak to barely detectable responses (subdominant) and even some that are only detectable in the absence of dominant epitopes (cryptic). Immunodominance in the antiviral response results from a combination of factors that include: the efficiency of antigen processing and presentation, the stability of peptide-MHC complexes, the overall number of peptide-MHC complexes, the available TCR repertoire, the competition between CD8+ clones for activation, viral evolution, or/and the concept of immunodomination in which T cells specific for some peptides suppress responses to other epitopes [7]–[11]. The hierarchies of T cell responses elicited by HIV infection are generally stable, e.g., specific HIV regions are consistently targeted across diverse ethnic groups despite their different HLA distributions and distinct infecting subtypes [12]. Although some dominant response patterns may be due to degrees of sequence conservation, recent analyses show that subdominant responses outside of highly conserved protein regions can significantly contribute to the *in vivo* control [13], [14]. Although no consistent correlation has been found between breadth of CTL responses and long-term viral control, it might prove advantageous for HIV-1 vaccines to overcome the immunodominance pattern seen in natural infection. Thus, detailed understanding of mechanisms that dictate HIV (non)-immunogenicity could help to induce alternative combinations of more evenly distributed immune responses as a means to optimize CTL responses in the vaccination setting.

As it is vital for the immune system to tolerate autologous structures for its proper regulation, any model of immunodominance should consider the potential impact of self/non-self discrimination. A breakdown in self-tolerance can lead to the onset of autoimmune diseases. It was proposed that self-tolerance might be infringed in autoimmunity by the activation of T cells directed against cryptic self-determinants [15], [16]: well-processed and -presented antigenic determinants are immunodominant, while poorly processed/presented ones curtail the activation of specific T cells and are thus cryptic. Then, certain conditions (e.g., addition of a cleavage site [17] or of inflammatory mediators [18]) can help circumvent self tolerance and thereby lead to autoimmunity. Viral or bacterial infections have also been suspected as triggering factors for autoimmune disorders, possibly through molecular mimicry and/or bystander activation of autoreactive cells. Indeed, as early as 1986, viral mimicry was suggested to play a role in the immune dysregulation seen in HIV infection, on the basis of i) HIV-1's structural similarities with MHC and various immune regulatory molecules [19]–[23] and ii) the presence of autoantibodies to HLA molecules [24]–[26].

We hypothesize that dominance profiles in the HIV-specific CTL responses reflect an inverse relationship between the similarity of an HIV epitope to the host proteome and its immunogenicity. The underlying rationale is that the immune system preferentially responds to antigenic sequences that are never or only sporadically encountered in the repertoire of self-antigens. To better understand the reported dominance patterns among responses to HIV-1 antigens, we first analyzed the extent of similarity between HIV-1 and the human proteome. Then, we present the first comprehensive investigation of the relationship between similarity to host and the profile of immune responses elicited against the whole HIV proteome in 314 HIV-infected individuals and against 944 overlapping peptides representing a broad range of individual HIV-1B Nef variants in 30 individuals. In doing so, we validated our hypothesis that HIV immunogenicity could be partially regulated by similarities to the host proteome.

## Results

### “Human-like” motifs in the HIV-1 proteome

We first identified HIV-1 segments with sequence identity (4-, 5- and 6-mers) or high similarity (9-mers) to human proteins by comparing all possible 4, 5, 6 or 9-mers derived from HIV-1 B consensus 2001 to the human proteome. Sequential overlapping k-mers were scanned using BLASTP [27]–[29] against a non-redundant library of human proteins to identify exact matches for 4, 5 and 6-mers while allowing up to 2 mismatches for 9-mers, since cross-reactivity drops markedly beyond 2 changes out of 9 amino acids (AA) (Heckerman, unpublished data).

Besides previously described similarities with MHC molecules [19]–[23], [30], numerous matches corresponded to human endogenous retrovirus (HERV) sequences. This was expected since endogenous retroviruses reflect past exogenous retroviral infections and are a large (2 to 8%) component of the human genome [31], with 950 full length reverse transcriptase (RT) [32] and 16 candidate genes potentially encoding functional Envelope (Env) proteins. The analysis also revealed HIV-1 similarities to proteins previously described as autoantigens, a conspicuous fact since the repertoire of autoantigens is thought to be limited to a few hundred [33]. The number of exact 4-mer HIV-1 matches against a set of 103 autoantigens (77.54 matches/1000AA) was significantly greater than matches against all proteins (54.56 matches/1000AA) (χ^2^ = 9.677). Segments in all HIV-1 proteins except Tat, Rev, and Vpr showed high similarities to various proteins of the NACHT-LRR (leucine-rich repeats) family, which have been implicated in apoptosis, inflammation and autoimmunity, in particular in the control of immune and inflammatory responses, and include key regulators of pathogen recognition [34]. Though their exact roles are not clearly defined, some NACHT-LRR have been genetically linked to immunological disorders [35], including immunodeficiency [36].

We next focused our search on nonamers, the typical length of epitopes presented by HLA class I molecules, in order to identify the longest “human-like” motifs. We identified 16 HIV oligomers considered similar to human proteins since they differed by 3 standard deviations from the similarity expected with randomized nonamers (Table 1). Of note, the HIV-1 consensus sequence showed only 2 mismatches with the 37-AA-long protein RAK [37]. However, based on virtual identity over 142 nucleotides to an HIV-1 isolate and lack of adaptation to human genome codon usage, the reportedly cancer-associated protein RAK is most likely an HIV contaminant (Learn, unpublished data). To account for HIV-1's extensive variability, the remaining 15 oligomers were compared to the full spectrum of HIV-1 sequences available in databases in order to identify further similarities. Briefly, the shared segments extended to one identical nonamer (in the protein Copine 5) and up to 11 identical AA out of 13 (RT of an HERV-K sequence).

**Table 1:** HIV-1 nonamers with high similarities to human proteins.

Significant 9-mers consensus 2001	Human Protein sequence	Human protein	HIV database seq. corresponding to human prot. seq.	HIV-I B consensus 2001 position	Identities (Positives)
LHPVHAGPI	L***n***PVHAGPIV	PTPRE		Gag 215–223	9/10 (10/10)
	LHP***G***HA***E***PIV	T cell leukemia Homeobox 1	LHP***V***HAEPI		8/10
EPFRDYVDRF	***V***PFRDYVDR	COPINE 5	AAV71076	Gag 291–300	8/9
	PFRDYVDR***S***		AAV53244		8/9
PDCKTILKA	HPDCKTI	DELTEX 4 homolog	AAV49378	Gag 328–336	7/7
NTPVFAIKKK	SP***W***NTPVF***V***IKKK	HERV	P***Y***NTPVFVIKKK	Pol 209–221	11/13
MTKILEPFR	MTKILEP***C***	Piwi-like 2	AAM74596	Pol 319–327	7/8
	***s***MTKIL***DS***F	SON DNA Binding P.	AAK35877		6/9 (7/9)
FKNLKTGKY	FKN***S***KTG	Str Spe Recognition P.	FKN***N***KT	Pol 501–508	6/7
VNIVTDSQYA	VNI***Y***TDSQYA	HERV	VNI***I***TDSQYA	Pol 648–657	9/10
	VN***T***VTD***DD***YA	Thrombospondin 3 precursor	VNI***V***TD***sE***YA		7/10
YIEAEVIPA	GYIEA***A***VIPAG	unnamed P.		Pol 798–806	10/11
	EIEAEV	p53 inducibleP.	AAV49469		6/6
NWRSELYKY	***H***NWRSELY	peroxysomal acylcoA thioestherase	CAA64162	Env 469–477	7/8
AAG…TVW	35/37	Cancer associated RAK		Env 516–572	35/37
DQGPQREPY	qRP–DQGPQR***P***P	carcinoma ass. P.	DQGPQR***A***P	Vpr 7–15	9/13 (10/13)
	DQGPQR***P***P	proline rich			7/8
PKTACTNCY	SQPK***s***AC***G***NCY	Zn finger P.	SQPK***t***AC***S***NCY	Tat 18–26	9/11 (10/11)
LAIVALVVA	ALVALVVA***P***L	Trypsin-domain P.	ALVALVVA***V***L	Vpu 7–15	9/10
	LAIVAL***A***V	small inducible cytokine 28			7/8
EELLKTVRL	EELLK***E***VRL	nucleolar RNA associated P.	EELLK***A***VRL	Rev10–18	8/9
	LLKTVRL***L***RLL		LLKTVRL***i***RLL		10/11
TVRERMRRA	TTVR***S***RMRRA	Rhomboid P.		Nef 15–23	9/10
IYSQKRQDI	TYSQK***F***QDI	Ig heavy chain variable region	TYSQK***R***QDI	Nef 101–109	8/9

The level of similarity to host proteins for these HIV nonamers differed by 3 standard deviations from the level of similarity found with randomized nonamers. Amino acid changes are in bold italics and lower case.

Finally, a spectrum of 182 HIV-1 B Env sequences dating from 1983 to 2005 was scanned for similarities to human proteins in order to identify whether HIV was adapting to become more or less host-like over time. Despite marked differences among individual HIV-1 sequences, there was no evidence of change in the degree of similarity to host proteins since the beginning of the widespread epidemic (data not shown).

### Inverse relationship between similarity of HIV to its host and its immunogenicity

**Figure 1:**
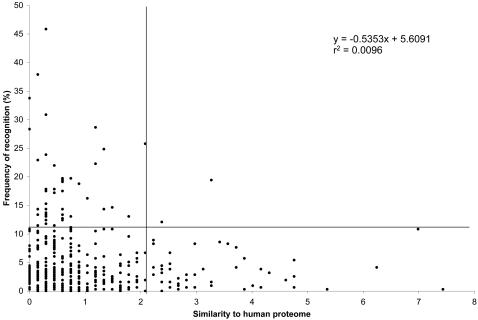
Frequency of recognition of HIV-1 B consensus 2001 peptides as a function of their similarity to human proteins. 410 peptides spanning the HIV-1 B consensus 2001 proteome were tested by ELISpot using PBMC from a cohort of 314 HIV-1 infected individuals. The vertical axis corresponds to the percentage of individuals who recognized a given peptide. The horizontal axis corresponds to the number of matches to the human proteome for each peptide. Matches were derived by dissecting the 410 peptides into overlapping 6-mers offset by one residue, and then scored against the RefSeq protein sequence database. Matches were normalized to account for the length of the starting peptide (ranging from 15–20 AA in length).

To investigate whether the similarity of HIV to its host participates in shaping the antiviral CD8+ T cell responses, we compared the similarity to human proteins of 410 HIV-1 peptides spanning the whole viral proteome to their frequency of recognition measured by IFN-γ ELISpot assays in 314 HIV-infected individuals [12]. We found a trend indicating an inverse relationship between the similarity to the host proteome and the ELISpot reactivity (Figure 1), consistent with our hypothesis that antigenic motifs scarcely represented in human proteins are more likely to be CTL targets for the host. The peptides that most frequently elicited a response presented less matches to human proteins, i.e., a low similarity to the host. Conversely, the peptides with high number of matches to human proteins more rarely elicited CTL responses. Numerous peptides were never or only marginally recognized in the population; hence our observation is only supported by a trend (r^2^ = 0.0096). Nonetheless, when Figure 1 is divided in 4 quadrants (based on the mean plus one standard deviation for the normalized similarity to the human proteome (x axis) and for the frequency of recognition by ELISpot (y axis)), the paucity of peptides in the top right quadrant (2 data points out of 410) emphasized that peptides with a high similarity to the host usually elicited CTL responses only in a small proportion of HIV-1 infected individuals. We randomized the relationship between the x and y axes and counted the number of times that both x and y axis values were superior to the mean plus one standard deviation (i.e., would fall in the top right quadrant). 1,000 randomizations were performed and there were 46 occurrences with no more than 2 data points meeting the criteria; hence, the dearth of data points in the top right quadrant found in our data would not be expected by chance (p = 0.046) and thus supported the hypothesis that peptides with high similarity to host proteins had a lower propensity to be recognized by the host CTL.

To lift the uncertainty in epitope specificity cast by using 18-mer peptides, 944 10mer peptides encompassing Nef were screened in 30 HIV-infected individuals using IFN-γ ELISpot assays. The 944 Nef 10-mers, akin to or slightly larger than the typical size of an HLA class I epitope, overlapped by 9 residues and represented a broad array of HIV-1 variants found in the population (Frahm et al., in preparation). 346 Nef peptides elicited a response in at least one patient while 598 did not. Peptides that were recognized had a significantly lower degree of similarity to human proteins than the 10-mers that were not reactive. Considering 9-mer matches (allowing 2 mismatches), the mean number of matches was 1.40 for the peptides eliciting a response compared to 3.16 for those that do not elicit any response (p = 0.0002). Similarly, for 5- and 6-mer exact matches, the mean number of matches were 55.75 and 3.09, respectively, for the peptides eliciting a response, compared to 107.51 and 6.02, respectively, for those that failed to elicit any response (p = 0.0002 and <0.0001, respectively). Since the peptides are overlapping, we did a cross-validation analysis to verify that overweighting the degree of similarity to humans or re-counting responses did not skew our results. We partitioned our data in 10 non-overlapping sets of peptides (each including from 88 to 102 peptides) and compared the similarity to human proteins to the ELISpot reactivity for each set. Although the p-values were affected (11 out of 30 were >0.10), ELISpot reactive peptides showed less similarity to human proteins than non-reactive ones.

**Figure 2:**
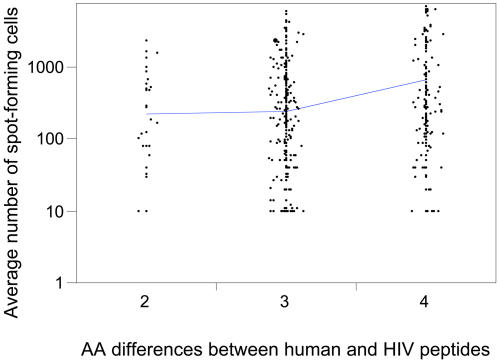
Magnitude of the ELISpot responses elicited by HIV-1 B Nef peptides as a function of their similarity to human peptides. The vertical axis corresponds to the mean number of spot-forming cells counted in ELISpot assays. The horizontal axis corresponds to Nef peptides that have 2, 3 or 4 differences with their closest human peptides.

Last, we analyzed the magnitude of ELISpot responses elicited by the Nef peptides stratified according to their similarity to their closest human peptides. The mean magnitudes (number of spot-forming cells) of the ELISpot responses were higher when the Nef peptides were more distant from their closest human peptides. When the Nef peptides had, respectively, 1, 2, 3 or 4 mismatches with their closest human peptides, the mean magnitudes were 0, 220.00, 237.99 and 654.18 respectively (Spearman correlation factor rho = 0.1911 and p-value = 3.2622e-09) (Figure 2).

### Multiple factors influence HIV-1 immunogenicity

The hypothesis of immunodominance being related to low similarity to self is beguiling but there are undoubtedly other concurring factors. Frahm et al. (2004) previously analyzed different parameters affecting a peptide's ability to elicit an immune response. ELISpot reactivity was favored by low peptide variability, low representation of forbidden AA (i.e., AA not generally found at the C-terminus of CD8+ T cell epitopes) and high proteasome cleavage likelihood scores. In addition, HIV regions enriched in CTL epitopes were shown to be more hydrophobic [38], [39]. In our genome-wide analysis, the relationship between peptide hydrophobicity and frequency of recognition was strongly supported only for Gag and Nef (data not shown). Overall, the effects of entropy and of forbidden residues were more pronounced than those of the degree of similarity to self, hydrophobicity, or disorder prediction score, as shown by a multivariate analysis: Y = 9.41, −0.38 (similarity), −5.22 (forbidden AA), +0.03 (hydrophobicity), −0.11 (disorder), −5.75 (entropy). In addition, it is important to note that the ratio of forbidden AA at the C-terminus of CD8+ T cell epitopes and the similarity to host proteins are positively correlated (p = 0.026): peptides highly similar to host proteins are not frequently recognized in ELISpot assays and also have a high ratio of forbidden AA, i.e., they are expected to be poorly presented.

**Figure 3:**
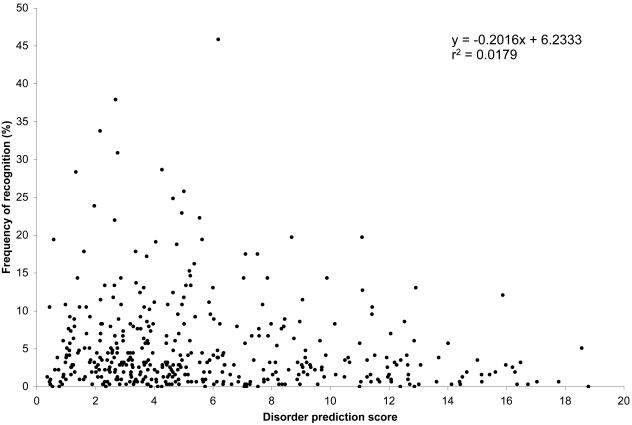
Frequency of recognition of HIV-1 B consensus 2001 peptides as a function of their intrinsic disorder prediction score. The vertical axis corresponds to the percentage of HIV-1 infected individuals that recognized each of the 410 peptides spanning the HIV-1 B consensus 2001 proteome. The horizontal axis corresponds to the disorder prediction score for each peptide, calculated using predictions of order/disorder made with the VSL1 predictor (PONDR®).

Lastly, we asked whether disorder/order of the peptides played a role in HIV immunogenicity. Disordered regions present a low sequence complexity with many repetitive elements coupled with a biased amino acid content deprived of bulky hydrophobic amino acids (that typically form the cores of folded globular proteins) while they are enriched in alanine, arginine, glycine, glutamine, serine, proline, glutamate and lysine that results in highly charged surfaces [40]; they are reported to bind to nucleic acid and form coiled-coils [41]. Different factors led us to take structural disorder into account: 1) many HIV segments showed similarity to autoantigens, which have a propensity for intrinsic disorder [41], 2) the presence of disorder in TY transposable element proteins suggests that it is a feature of retroviruses, since TY elements in the yeast genome result from retroviral infection [42], and 3) Tat was recently shown to be unfolded in its native state [43]. We found that most HIV proteins presented regions of intrinsic disorder and that highly disordered peptides did not elicit broad CTL responses while highly recognized peptides showed low peptide disorder (Figure 3).

## Discussion

Our data highlight a likely influence of the similarity to human sequences in shaping the host's immune response to HIV-1. We found that HIV-1 infected individuals seldom recognized the most “human-like” peptides, while the peptides that were frequently recognized showed a low similarity to the host. The relationship between HIV-1B similarity to host proteins and its immunogenicity was evidenced in 2 ways by analyzing CTL responses against i) 410 consensus peptides representing the whole HIV-1 B proteome in a cohort of 314 individuals and ii) 944 variant Nef peptides in 30 individuals. Furthermore, the more a Nef peptide was different from its closest human peptide, the stronger the immune response it elicited. These results, stemming from consensus and circulating HIV variants, support HIV's degree of similarity to host as a mechanism contributing to the intrinsic hierarchical profile of HIV-specific CD8+ T cell responses seen across different cohorts and host ethnicities [12], [44]–[48]. However, similarity to host proteins affect only a limited proportion of HIV-1 peptides and is unlikely to impact the immune evasion that plays a critical role in disease progression in HIV infected individuals. The relationship between HIV peptides' similarity to self and immunogenicity is nonetheless worthwhile understanding in the absence of clear correlates between breadth/magnitude of CTL responses and viral containment.

Although the extent of similarity between HIV and human proteins is relatively limited, a variety of segments are shared between HIV and host proteins, particularly proteins involved in host immunity, underlining a potential role of HIV-1's similarity to host in the interference with effectors of the immune system. For example, it could be expected that HIV/HERV mimics would have reduced immunogenicity, as negative selection in the host should have largely eliminated reactive T cell populations. Indeed, HERV expression was detected in the thymus, where immune tolerance toward self is maintained via central tolerance; thymocytes with a high affinity for self-antigens are deleted, thus the remaining thymocytes (the future CD4+/CD8+ T cells) are likely to have a low affinity for HERV-like antigens. It should be noted that high similarity to HERV was found outside of the reverse transcriptase active site (where similarities between enzymes are expected). In addition, frequent similarities between HIV and autoantigens suggest the possibility of cross-reactive responses through molecular mimicry. This may help explain why HIV patients appear to be more prone to develop autoantibodies, in particular against cardiolipin, ribonucleoproteins, smooth muscle, platelets or cryoglobulinemia [49]–[52]. Moreover, two broadly neutralizing HIV-1 monoclonal antibodies were found to be autoreactive against the self-protein cardiolipin [53], and antibodies cross-reacting with HIV-1 p24 are found in 30% of patients with the autoimmune Sjogren's syndrome [54]. Although auto-reactive responses are usually neither harmful nor do they lead to overt autoimmune diseases in most patients, HIV antigens depleted of self-mimicking elements may represent safer vaccines.

By showing a relationship between peptide similarity and immunodominance we validated low similarity to the host proteome as a concurring factor in the modulation of the pool of epitopic sequences, with a potential role in discriminating immunodominant from cryptic peptides. Nonetheless, it must be added that the molecular basis of immunogenicity is the outcome of numerous interacting factors, among which we studied structural disorder. Intrinsically disordered regions are protein segments which lack a fixed tertiary structure (i.e., they are partially or fully unfolded); they are involved in many biological functions including cell signaling, regulation, molecular recognition and other interactions with proteins and nucleic acids [55]. Thus, the intrinsic disorder of HIV segments, and their associated conformational flexibility and potentially proteolytic lability could impact the skewed immune response seen in HIV infection. It is possible that native disorder could be involved in allowing the virus to evade immune detection, since disordered regions would generally correspond to poor activators of B cells, with few B cells able to bind with high affinity to disordered determinants. Although structural similarity between virus and host could also impact HIV-specific immune responses, our analysis only addressed sequence identity and MHC-1 responses, since investigating the impact of structural similarity between virus and host is currently hampered by the enormous computing power required and the limited knowledge of the structural conformation of epitopes.

Since it was recognized that some conformational structures are favored for HLA binding [56], immunogenicity profiles have been analyzed with respect to several physico-chemical proprieties, including hydrophobicity. The presence of HLA binding motifs or processing signals, together with the absence of AA that negatively impact peptide cleavage, have been previously associated with the propensity to elicit a response [38], [39]. We found a positive relationship between the similarity to host proteins and the proportion of AA that are typically absent from the C-terminus of CD8+ T cell epitopes. Thus, peptides with a high similarity to self are expected to be poorly processed and presented, and thereby are likely to be cryptic determinants. Indeed, our data show that those peptides are typically marginally targeted in the population. Moreover, since we identified similarities between HIV and some autoantigens, we can also envisage that the similarity of HIV epitopes with host proteins could reverse the cryptic nature of these self-epitopes and thereby lead to the initiation of autoreactivity. Together, our data corroborate and extend the model proposed by Moudgil and Sercarz, which puts forth the gradient of presentation efficiency as a basis for immunodominance [16].

These findings illustrate how numerous factors can intersect to establish an immunodominance hierarchy and show that high similarity to the host proteome hampers peptide immune reactivity. Thus, removing host mimics from vaccine constructs could be a crucial step toward designing not only safer but also more efficacious HIV vaccines. Due to the lack of viral peptides that are simultaneously similar to the host and strongly immunogenic, we suggest design of HIV vaccine candidates using non-self discrimination as a molding force in generating peptide immunogenicity. Crafting more potent vaccine candidates hinges upon the accurate understanding of the molecular mechanisms involved in peptide immunogenicity, and particularly upon a deeper insight into immunodominance.

## Materials and Methods

### Database Searches and Sequence Analysis

Analyses of the degree of similarity between HIV-1 and the human proteome were conducted using the HIV-1 subtype B consensus sequence of 2001 available at the HIV immunology database (http://www.hiv.lanl.gov/content/hiv-db/PEPTGEN/2001.html). The 9 HIV-1 protein sequences were dissected into overlapping 4, 5, 6 or 9-mers offset by 1 residue, which were probed for sequence similarity against a protein database representing the human genome (27,606 proteins corresponding to 14,236,486 AA). The non-redundant human protein collection was retrieved from the RefSeq database, available at the NCBI website (http://www.ncbi.nlm.nih.gov/entrez/query.fcgi?db = Protein). Searches against the human library were run using ViroBLAST (Modification of the Basic Local Alignment Search Tool available at: http://indra.mullins.microbiol.washington.edu/)[29], with parameters set for short, nearly exact match searches. The 4, 5 and 6-mers were probed for exact matches with human protein sequences, while up to 2 mismatches were allowed for 9-mers. Database hits were curated manually based on sequence annotation and reference to the literature; initial hits caused by contamination of the RefSeq database by non-human sequences (corresponding mostly to viral or bacterial elements) were removed from further analyses. Similar analyses were conducted for i) 182 Env protein sequences (C2-V5 region) dating from 1983 to 2005 and ii) 944 10-mer-sequences spanning the Nef protein.

### Immunological analysis

IFN-γ ELISpot assays were performed using overlapping peptides, as previously described [12]. They include 410 peptides (usually 18-mers–varying from 15 to 20 amino acids in length-overlapping by 10 AA) spanning the entire HIV-1 subtype B consensus 2001 proteome, and were tested using PBMC from 314 HIV-1 infected individuals. A set of 944 peptides (10-mers overlapping by 9 AA) corresponding to HIV-1 subtype B Nef variants was tested using PBMC from 30 HIV-1 infected individuals (Frahm et al, in preparation). For each peptide, the numbers of matches found in the similarity analyses (for all k-mers studied) were tallied and compared to the peptide's frequency of recognition in IFNγ ELISpot assays.

### Multivariate analysis

Different parameters were analyzed for the 410 overlapping 18-mer-peptides. The average Shannon entropy scores for all peptides are available at the HIV immunology database (http://hiv-web.lanl.gov/content/immunology/hlatem/study1/peptides.html). The frequency of “forbidden” amino acids and cleavage scores were determined for each peptide as described [12]. A disorder score was calculated using predictions of order/disorder made with the VSL1 predictor available at the PONDR® web-site (http://www.pondr.com) [57]. The PONDR® Predictors of Naturally Disordered Regions are feed-forward neural networks that use primary sequence data (generally windows of 21 amino acids). The predictions are smoothed over a sliding window of 9 amino acids: a residue is considered disordered if it exceeds a threshold of 0.5. The composite predictor VSL1 was developed to improve the prediction performance on short disordered regions (<30 residues); the VSL1 implementation showed the best prediction performance in an independent assessment of 20 order/disorder predictors [58]. Stepwise multiple-regression analysis was carried out to model the contributions of these variables to predicting the frequency of recognition for each peptide.

### Statistical analysis

Statistical analyses were done using JMP® version 5.1.2. Correlations were considered statistically significant when p was <0.05.

Randomization analysis of the frequency of recognition of HIV-1 B consensus 2001 peptides as a function of their similarity to human proteins. The values of the x axis (normalized similarity to the human proteome) were randomized. The Null distribution corresponds to the number of times when both x and y axes values were greater than their means plus 1 standard deviation. 1000 randomizations were performed. The number of occurrences (with both x and y axes values greater than their means plus 1 standard deviation) were counted and compared to the observed number of occurrences.
